# General Grant’s Condition

**Published:** 1885-04

**Authors:** Frank Abbott


### GENERAL GRANT'S CONDITION.

There having appeared in the medical journals and the daily papers so many statements in reference to the case of General Grant, the condition of his teeth, the cause of their removal, etc., etc., I am induced to give to the profession and the public, through these columns, the facts, as far as they have come under my observation and treatment. On the 8th of November, 1884, General Grant consulted me by advice of his physician, Dr. Fordyce Barker, in reference to the advisability of having the right upper first molar extracted, stating that he had for a week or ten days been suffering from great pain on that side of his head and face, and that his physician had advised the removal of this tooth, as it was, in his opinion, the cause of the trouble. Upon examination I found the tooth dead, with an abscess at the apex of the anterior buccal root. It was considerably projected from its original position, and the neck and perhaps one-half the length of the roots were covered with tartar. I at once coincided with Dr. Barker, that this was probably the cause, in a measure at least, of the neuralgia, and advised its immediate removal, and this was accordingly done. The relief from severe localized pain was prompt and highly satisfactory to the General, as well as to myself. I made no further examination of his teeth at that time, as he expressed a desire to make appointments with me in a few days, for doing what should be found necessary for the health and comfort of his mouth, and for the reason that he wished time to recuperate from the distressing pain and sleeplessness from which he had so long been suffering. On the 14th of the same month I again saw him, and carefully examined his remaining teeth. The first to particularly attract my attention were the second and third right superior molars, posterior to the opening from which I had removed the first molar. Both of these teeth were projected from a quarter to three-eighths of an inch, being drawn from their original positions; the necks and at least two-thirds of the length of their roots were thickly coated with a dark brown or black tartar, the sockets in consequence being almost entirely absorbed, while the teeth were so loose that they might easily have been removed with the ancient "lead forceps." In addition to this the second molar was very badly decayed, the side wall broken down, and the pulp dead. The surrounding gum was greatly irritated, the inflammation extending to the roof of the mouth, and back into the fauces. Finding these teeth in so deplorable a condition, and there being no antagonizing teeth, I advised their immediate removal, believing, as I did then, and do now, that they had more or less agency in at least aggravating the lesion which then (and had for five months) existed in his throat and upon his tongue. He consented, and I at once removed the teeth. I then removed some of the tartar from other teeth in his mouth, and dismissed him. I again saw him on the 17th of the same month, at which time I thoroughly cleansed his teeth and put in some fillings, all that was necessary to do upon the teeth remaining in his mouth. How long these teeth had been in the general condition in which I found them I am unable to say, but probably (and I judge from all the conditions described) it had been many years. If we take into consideration the highly irritable condition of that region of his mouth, the extension of the inflammation into the vault and down that side of his throat, the rough and ragged surfaces of a broken tooth, and the tartar incrustations, against which the side of his tongue was almost constantly rubbing, I believe that I am quite justified in concluding that here may have been a factor, at least, in the localization of the painful disease from which the General is now suffering, instead of attributing it altogether to the habit of smoking, which seems to be the prevailing opinion, at least among the laity.

The motive that prompts me to write this article is a desire that the case may stand as a solemn warning to all those *advancing in years*, against the tolerance of any rough surfaces (such as are here described) in the mouth, by which the tongue or other tissues may be kept in a state of constant irritation, and that an instructive instance may be laid before my brother dentists.

March 20, 1885. FRANK ABBOTT, M. D.

